# Dynamic changes of SETD2, a histone H3K36 methyltransferase, in porcine oocytes, IVF and SCNT embryos

**DOI:** 10.1371/journal.pone.0191816

**Published:** 2018-02-15

**Authors:** Yun Fei Diao, Tao Lin, Xiaoxia Li, Reza K. Oqani, Jae Eun Lee, So Yeon Kim, Dong Il Jin

**Affiliations:** 1 Institute of Special Animal & Plant Sciences, Chinese Academy of Agricultural Sciences, Changchun, China; 2 Department of Animal Science & Biotechnology, Research Center for Transgenic Cloned Pigs, Chungnam National University, Daejeon, Republic of Korea; Macau University of Science and Technology, MACAO

## Abstract

SETD2 (SET domain containing protein 2) acts as a histone H3 lysine 36 (H3K36)-specific methyltransferase and may play important roles in active gene transcription in human cells. However, its expression and role in porcine oocytes and preimplantation embryos are not well understood. Here, we used immunofluorescence and laser scanning confocal microscopy to examine SETD2 expression in porcine fetal fibroblasts, oocytes, and preimplantation embryos derived from in vitro fertilization (IVF), parthenogenetic activation (PA), and somatic cell nuclear transfer (SCNT). In porcine fetal fibroblasts, SETD2 expression was detected in interphase cells, but not in M (mitotic)-phase cells. The SETD2 signal was observed in non-surrounded nucleolus (NSN)-stage oocytes, but not in surrounded nucleolus (SN)-, metaphase I (MI)-, or metaphase II (MII)-stage oocytes. The SETD2 signal was detectable in sperm, and undetectable immediately after fertilization, detectable at the 2-cell stage, and peaked at the 4-cell stage of IVF embryos in which porcine embryonic genome is activated. Similar to the pattern found in IVF embryos, the SETD2 signal was absent from PA embryos at the 1-cell stage, but it was detected at the 2-cell stage and thereafter maintained to the blastocyst stage. Interestingly, unlike the IVF and PA embryos, the SETD2 signal was detected throughout the development of SCNT embryos, including at the 1-cell stage. These data suggest that SETD2 may be functional for embryonic gene transcription in porcine preimplantation embryos. It is further speculated that the aberrant expression of SETD2 at the 1-cell stage of porcine SCNT embryos may be a factor in the low efficiency of cloning in pig.

## Introduction

Histone modifications, including acetylation, phosphorylation, methylation, and ubiquitination, etc., are connected with many fundamental biological processes in cells [1]. A great deal of research has focused on the relationship between histone acetylation changes and embryonic genome activation (EGA) in mammalian embryos. Indeed, the developmental capacity of embryos derived from somatic cell nuclear transfer (SCNT) can be dramatically improved in many species by histone deacetylase inhibitor treatment [2–6]. A recent study indicated that histone H3K36 tri-methylation (H3K36me3) is developmentally regulated and may act as a histone mark of EGA for pig in vitro fertilized (IVF) embryos, but becomes aberrant during the nuclear reprogramming of porcine SCNT embryos [7], potentially accounting (at least in part) for the low developmental efficiency of cloning. Thus, studies are needed to examine the underlying mechanism and regulation of H3K36me3 during the development of SCNT embryos.

Histone methylation plays a key role in eukaryotic transcriptional regulation [8], and histone methyltransferases with distinct functions have been reported. For instance, histone H3K27 methylation mediated by the EED-EZH2 complex has been associated with gene silencing [9] and X chromosome inactivation [10], whereas histone H3K9 methylation regulated by SUV39H1 has been correlated with heterochromatin organization [11] and X chromosome inactivation [12]. SETD2 (also known as HYPB, HSPC069, Hset2, or KMT3A) is the histone H3K36-specific methyltransferase identified to date [13]. It is recruited to sites of active transcription by an interaction with hyper-phosphorylated RNA polymerase II [8], suggesting that the SETD2 protein may play a critical role in transcriptional regulation. In mice, SETD2 is responsible for virtually all global and transcription-dependent H3K36me3 [14]. In human [13] and zebrafish [15], SETD2 acts as the ortholog of yeast SETD2. SETD2 plays important roles in alternative splicing, the repression of intragenic transcripts, chromatin accessibility, and the regulation of DNA mismatch repair [16–18]. SETD2-dependent histone-H3K36 tri-methylation (H3K36me3) is required for embryonic vascular remodeling [13], homologous recombination repair, and genome stability [19]. Knockdown of SETD2 in murine fibroblasts was associated with the loss of H3K36me3, indicating that SETD2 is a specific enzyme for H3K36me3 [14].

Although some biochemical mechanisms for the actions of SETD2 have been reported in mouse and human, no previous published study has examined the transcriptional regulation of SETD2 in mammalian oocytes and preimplantation IVF or SCNT embryos. Here, we investigated the dynamic changes of SETD2 in an effort to uncover the mechanisms responsible for reprogramming the gene expression patterns of oocytes and preimplantation embryos derived from IVF and SCNT in pig.

## Materials and methods

### Ethics statement

All of the experimental procedures used in this study were approved by the Institutional Animal Care and Use Committee of Chungnam National University (CNU-00628).

### Chemicals

All chemicals were obtained from Sigma Chemical Co. (St. Louis, MO, USA) unless otherwise indicated.

### Preparation of porcine fetal fibroblasts

Porcine fetal fibroblasts were isolated from fetuses at day 35 of gestation. Briefly, a pregnant sow was euthanized approximate 35 day after fertilization by intravenous injection of pentobarbital, and the entire uterus was removed for acquisition of fetuses. After fetuses were rinsed with PBS several times, the brain, internal tissues, and limbs were removed, and the remaining tissues were cut into small pieces with fine scissors. The minced tissues were incubated for 10 min at 38.5°C with 0.05% trypsin and 0.5 mM EDTA (15050–065; Gibco, Carlsbad, CA, USA), and the resulting suspension was centrifuged at 500 rpm for 10 min. The cell pellet was resuspended and cultured in DMEM supplemented with 75 μg/mL penicillin G, 50 μg/mL streptomycin, 5% (v/v) fetal bovine serum (FBS; Gibco, 16000–044) and 5% (v/v) fetal calf serum (FCS; Gibco, 26010–074). All cells were cryopreserved upon reaching confluence. Cells from passages 3–8 were used for experiments.

### Porcine oocyte collection and in vitro maturation

Pig ovaries were obtained from a local abattoir (NH Livestock Cooperation Association, Nonsan City, Chungnam Province, Korea) where we had acquired permission, kept at 35°C in PBS supplemented with 50 μg/ml streptomycin sulfate and 100 IU/ml penicillin, and transported to the laboratory within 3 h. Cumulus-oocytes complexes (COCs) were aspirated from antral follicles (3 to 6 mm in diameter) using a 10-ml disposable syringe fixed with an 18-gauge needle. Oocytes with a uniform ooplasm and more than three layers of cumulus cells were selected for in vitro maturation (IVM, Figure A in S1 File). For IVM, ~ 50 COCs in 500 μl maturation medium were cultured in each well of a four-well multi dish, at 38.5°C in saturated-humidity air containing 5% CO_2_. The maturation medium used for porcine oocyte maturation was TCM 199 supplemented with 3.5 mM D-glucose, 0.57 mM L-cysteine, 0.91 mM sodium pyruvate, 75 μg/ml penicillin, 50 μg/ml streptomycin, 10 ng/ml epidermal growth factor (EGF), 10 IU/ml pregnant mare serum gonadotropin (PMSG), 10 IU/ml human chorionic gonadotropin (hCG), and 10% porcine follicular fluid. After 22 h IVM, the COCs were washed and transferred to the same maturation medium without hormones for further culture.

### Embryo production by in vitro fertilization

In vitro fertilization (IVF) of oocytes was carried out as described previously by Lin *et al*. [20]. Briefly, matured cumulus-free oocytes were washed three times in modified Tris-buffered medium (mTBM) containing 113.1 mM NaCl, 3 mM KCl, 7.5 mM CaCl_2_, 11 mM glucose, 20 mM Tris, 2 mM caffeine, 5 mM sodium pyruvate, and 2 mg/mL BSA. After washing, groups of 10 oocytes were transferred into 50-μl droplets of mTBM and covered with warm mineral oil in a 35 × 10 mm Petri dish at 38.5 °C in 5% CO_2_. A fresh semen sample was obtained each week from the Darby Pig Artificial Insemination Center (Yeongi-gun, Korea). Each semen sample was washed three times by centrifugation at 1000 g for 3 min in Dulbecco’s phosphate buffered saline (DPBS) supplemented with 0.1% BSA. After the last washing, the sperm pellet was resuspended in mTBM at 1×10^6^ cells/ml, and 5 μl of the sperm suspension was added to a 50-μl mTBM droplet containing 10 oocytes. The oocytes were co-incubated with the sperm for 6 h at 38.5°C in a humidified atmosphere containing 5% CO_2_. The gametes were washed repeatedly with a fine glass pipette to remove attached sperm from the zona pellucida, and then cultured in 50 μl of PZM-3 containing 0.3% (mg/ml) BSA. The day of IVF was designated as day 1; cleavage and blastocyst formation were evaluated on days 3 and 7, respectively.

### Embryo production by parthenogenetic activation

For parthenogenetic activation (PA), cumulus-cell-free oocytes were washed three to five times with an activation solution (0.3 M D-mannitol, 0.1 mM MgSO_4_, 0.05 mM CaCl_2_, and 0.01% PVA). Electrical activation was induced with a 30-μs double-direct current pulse (DC) of 1.1 kV/cm, using an Electro Cell Manipulator 2001 (BTX, San Diego, CA, USA). After stimulation, the embryos were washed three times and transferred into 500 μl of culture medium (PZM-3 plus 3% BSA) in a four-well multi dish, covered with mineral oil, and incubated at 38.5°C in a 5% CO_2_ atmosphere. The day of PA was designated as day 1; cleavage and blastocyst formation were assessed at days 3 and 7 after activation, respectively.

### Embryo production by somatic cell nuclear transfer

For SCNT (Figure B in S1 File), we conducted nuclear transfer, fusion, and cytoplast activation as previously described [21]. Briefly, enucleation was carried out in PZM-3 supplemented with 0.3% BSA and 7.5 μg/mL CB at 38.5°C. Cumulus-free oocytes were enucleated by aspiration of the first polar body and adjacent cytoplasm with an enucleation needle. To examine the enucleation rate, a portion of the enucleated oocytes was stained with 5 μg/mL Hoechst 33342 and examined under a fluorescence microscope. After enucleation, injection was performed using porcine fetal fibroblasts. A donor cell was transferred into the perivitelline space of an enucleated oocyte. The reconstructed embryos were simultaneously fused and activated with two DC pulses of 1.1 kV/cm for 30-μsec per pulse (BTX Electro Cell Manipulator) in 0.3 M mannitol medium containing 1.0 mM CaCl_2_·H_2_O, 0.1 mM MgCl_2_·6H_2_O, and 0.5 mM HEPES. The oocytes were washed with PZM-3 containing 3% BSA, transferred into 50-μl micro drops of in the same culture media, covered with mineral oil, and incubated at 38.5°C in a 5% CO_2_ atmosphere. The day of SCNT was designated as day 1; cleavage and blastocyst formation were assessed at days 3 and 7 after activation, respectively.

### Immunofluorescence staining and quantification analysis

Porcine fetal fibroblasts at passage 5 were seeded onto coverslips in six-well dishes containing 3 ml/well cell culture medium (DMEM supplemented with 10% FBS) and grown to 90% confluence. Porcine GV, MI, and MII oocytes and 1-cell, 2-cell, 4-cell, 8-cell, and blastocyst-stage embryos derived from IVF, PA, and SCNT were obtained for immunofluorescence staining. Samples were washed in PBS containing 0.1% PVA (PBS-PVA), and then fixed in paraformaldehyde (2% in PBS-PVA) for 10 min. After another wash with PBS-PVA, the samples were permeabilized with 0.5% (v/v) Triton-X 100 and 100 mM glycine in PBS-PVA for 30 min, and then blocked with 3% BSA and 0.3% Triton-X 100 in PBS for 30 min. After two 10-min washes in PBG (PBS containing 0.5% BSA and 0.1% gelatin), the samples were incubated with primary antibodies (1:200) against H3K36me3 (Tri-methyl K36; 9763, Cell Signaling) or SETD2 (SET domain containing protein 2; ab69836, Abcam) at 4°C overnight. The samples were then washed in PBG for 20 min and then reacted with secondary antibodies (bovine anti-rabbit IgG-FIFC, 1:200, sc-2365, Santa Cruz Biotechnology) in the dark for 1h. Negative control embryos were performed as described above, but without any primary antibody. The samples were washed twice with PBG (10 min per wash), mounted on glass slides using Vectashield mounting medium with DAPI, and examined with a Zeiss laser-scanning confocal microscope equipped with x 20 or 40 objectives and running the Zeiss LSM Image Browser software (Zeiss). To improve the contrast with the green fluorescence, the color of the DAPI counterstain was changed from the original blue to red in all figures. Fluorescence intensity was assessed by analyzing the micrographs with the Image J software (National Institutes of Health, Bethesda, MD, USA) after background subtraction, as described previously [7]. The same instrument settings were used throughout our fluorescence intensity analysis, enabling us to analyze data from replicates.

### Statistical analysis

All data were subjected to one-way ANOVA using the SPSS 17.0 software (SPSS Inc., Chicago, USA), followed by Fisher’s protected least significant difference (LSD) test. At least three replicates were performed for each experiment. Bars representing least squares show the standard error for each group. P < 0.05 was considered significantly different.

## Results

### SETD2 status in porcine fetal fibroblast cells

**Fig 1:**
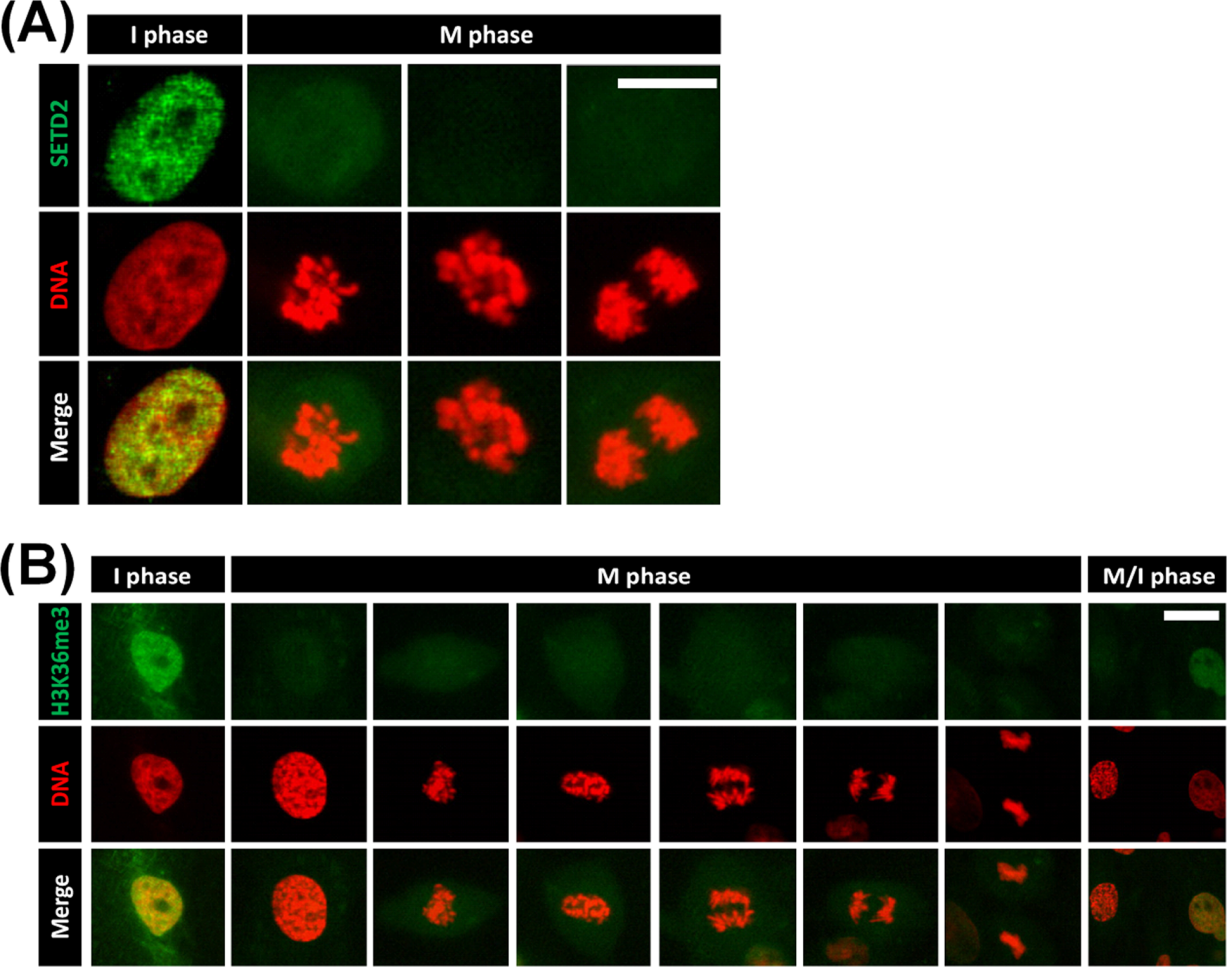
SETD2 and H3K36me3 status in porcine fetal fibroblast cells of different phases. Porcine fetal fibroblast cells were immunostained with primary antibodies against SETD2 (A) or H3K36me3 (B), followed by an FITC-conjugated secondary antibody (green). DNA was counterstained with DAPI, and the DAPI signal was colorized to red for better visualization. In the merged images, the orange color indicates areas in which SETD2 or H3K36me3 colocalize with DNA. I and M phase indicate interphase and mitosis phase, respectively. Scale bar = 30 μm.

We first investigated the intranuclear localization of SETD2 in porcine somatic cells. Immunocytochemistry with a specific antibody against SETD2 revealed that the fluorescence signals of SETD2 could be detected in interphase cells, but not M (mitosis)-phase cells (Fig 1A). The localization pattern of H3K36me3 was similar to that of SETD2 in porcine somatic cells: H3K36me3 exhibited a very clear signal in interphase cells, but was not detected in M-phase cells (Fig 1B).

### Changes of SETD2 status during porcine oocyte meiotic maturation and preimplantation development of PA embryos

**Fig 2:**
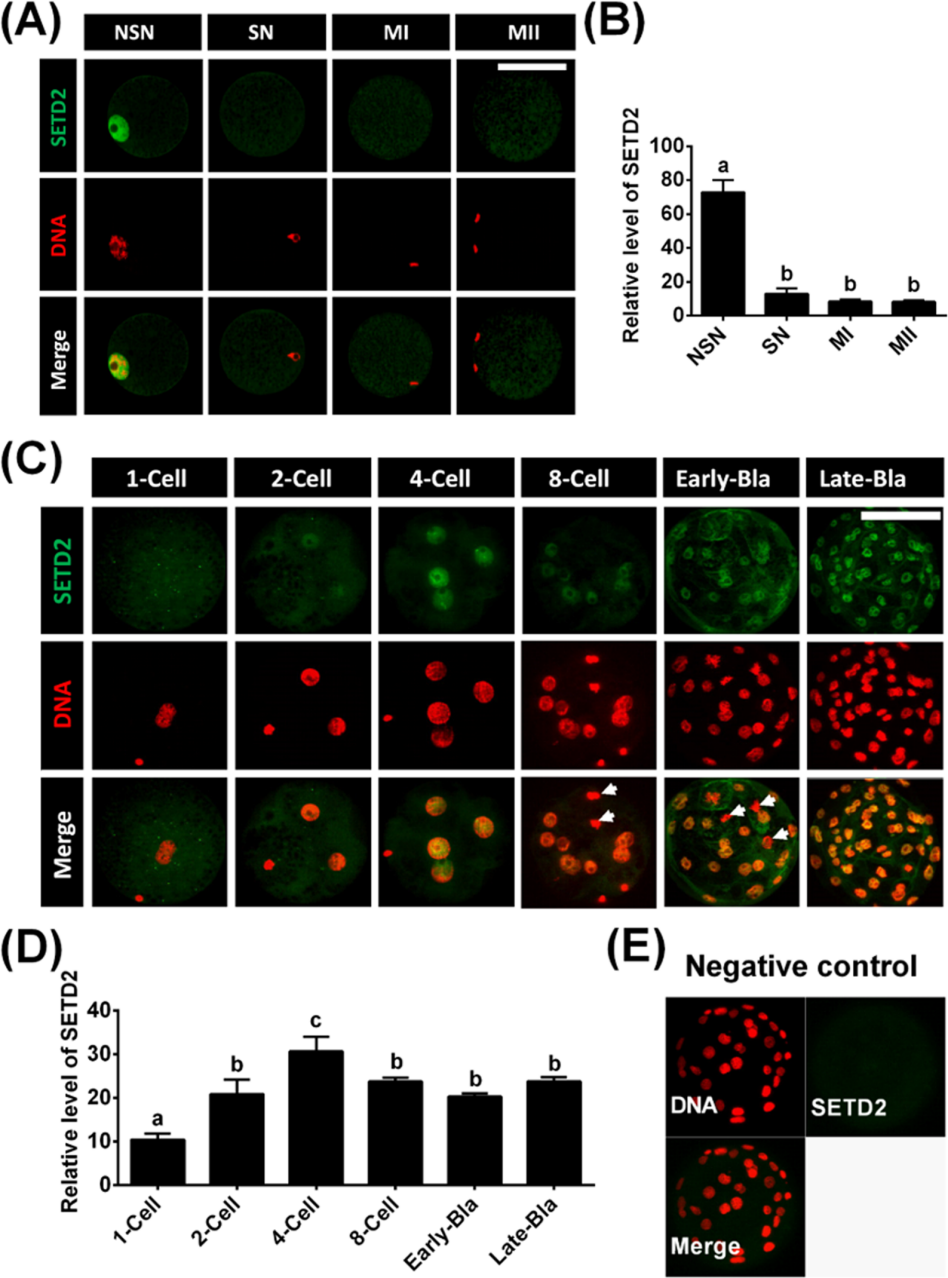
Changes of SETD2 status in porcine oocytes and PA-derived embryos. (A) Porcine oocytes were immunostained with the anti-SETD2 antibody followed by a FITC-conjugated secondary antibody (green). The DNA was counterstained with DAPI (colored red). NSN (non-surrounded nucleolus) and SN (surrounded nucleolus) indicate oocytes at germinal vesicle (GV) stages. MI and MII indicate oocytes at the stages of metaphase I and II, respectively. (B) Relative intensities of fluorescence signals from SETD2. (C) Porcine preimplantation embryos derived from PA were immunostained with anti-SETD2 antibody (green), while DNA was counterstained with DAPI (colored red). Arrows indicate M-phase blastomeres in 8-cell and early blastocyst stage embryos. Early and Late-Bla indicate early blastocysts and late blastocysts, respectively. (D) Relative intensities of fluorescence signals obtained for SETD2 in porcine PA embryos. (E) Negative control embryos were stained only with the secondary antibody. Total of 22 oocytes and 31 PA embryos was analyzed in triplicate in this study. ^a, b, c^ Least-squares with different superscripts are significantly different (P < 0.05). Scale bars = 100 μm.

To investigate the dynamics of SETD2 during meiotic maturation, we immunostained porcine oocytes derived from different stages. In germinal vesicle (GV) oocytes, SETD2 expression was always detected in non-surrounded nucleolus (NSN)-stage oocytes (100%, 12 of 12), whereas little (9.1%, 1 of 11) or no such signal (90.9%, 10 of 11) was detected in surrounded nucleolus (SN)-stage oocytes (Fig 2A and 2B). Similar to our findings in SN-stage oocytes, no signal was observed in metaphase I (MI)-stage (100%, 12 of 12) or metaphase II (MII)-stage (100%, 12 of 12) oocytes (Fig 2A and 2B). We also examined the SETD2 status in PA embryos using an antibody against SETD2. As shown in Fig 2C and 2D, the SETD2 signals in PA embryos were first detected at the 2-cell stage, peaked at the 4-cell stage, and were maintained to the blastocyst stage. In contrast, little or no such signal was observed in 1-cell-stage embryos.

### SETD2 status in porcine IVF embryos

**Fig 3:**
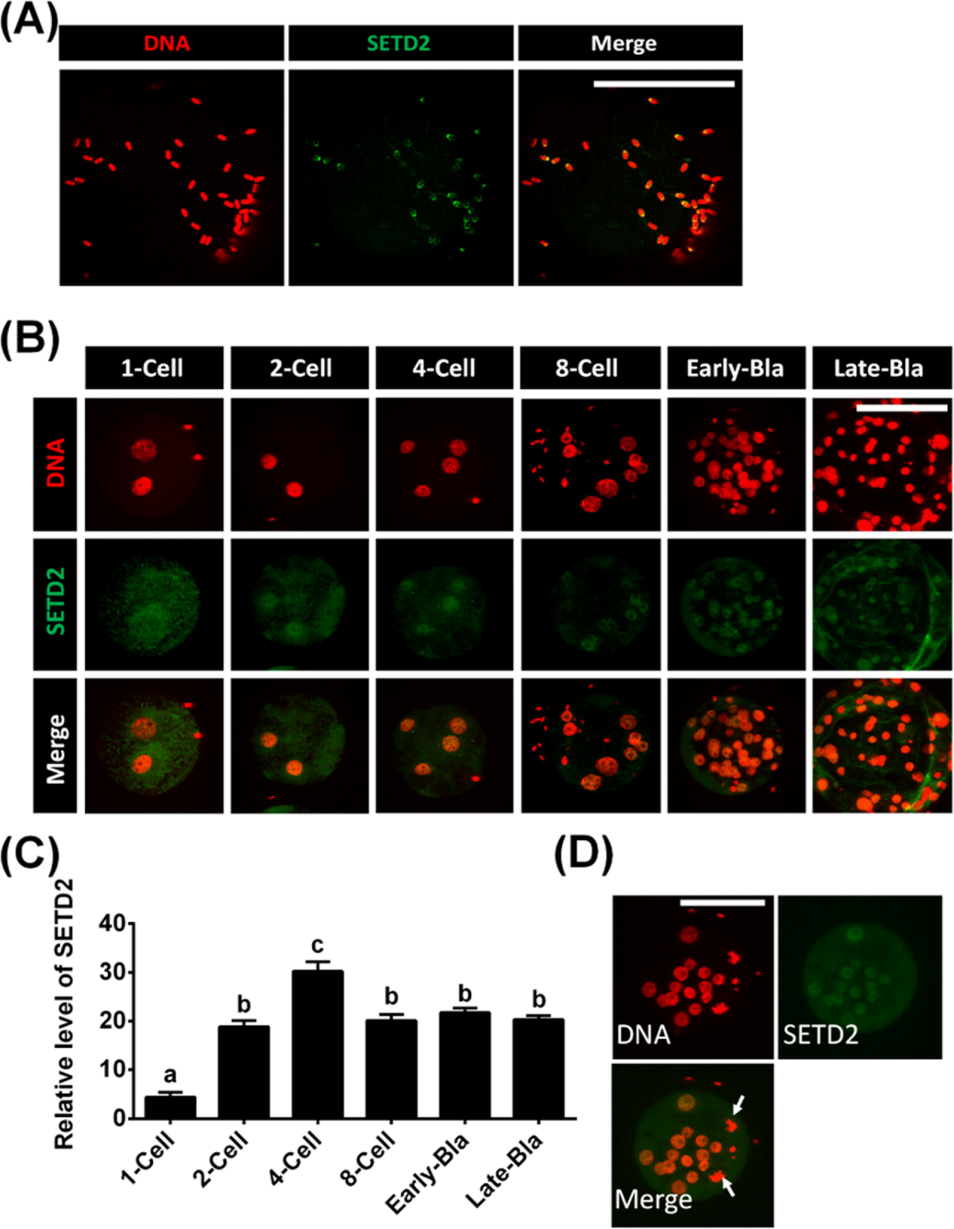
SETD2 status in porcine IVF embryos. Porcine sperm (A) and IVF-derived embryos (B) were immunostained with the anti-SETD2 antibody, which was visualized with an FITC-conjugated secondary antibody (green). DNA was counterstained with DAPI (colored red). (C) Relative intensities of fluorescence signals obtained for SETD2 in porcine IVF-derived embryos. Total of 31 IVF embryos was analyzed in triplicate in this study. (D) A representative IVF embryo showing blastomeres at M phase (arrows). Early and Late-Bla indicate early blastocysts and late blastocysts, respectively. ^a, b, c^ Least-squares with different superscripts are significantly different (P < 0.05). Scale bars = 100 μm.

To examine the dynamic changes of SETD2 in porcine IVF embryos, we immunostained porcine sperms and IVF embryos of different stages. SETD2 was detected in porcine sperms (Fig 3A), but it was no longer detected in male or female pronuclei (1-cell-stage embryos) after fertilization (Fig 3B). The signal became detectable once more at the 2-cell stage, peaked at the 4-cell stage, and was maintained until the blastocyst stage (Fig 3B and 3C). We also found that little or no SETD2 was detected in M-phase blastomeres of preimplantation embryos derived from IVF (Fig 3D).

### Dynamic changes of SETD2 status in porcine SCNT embryos

**Fig 4:**
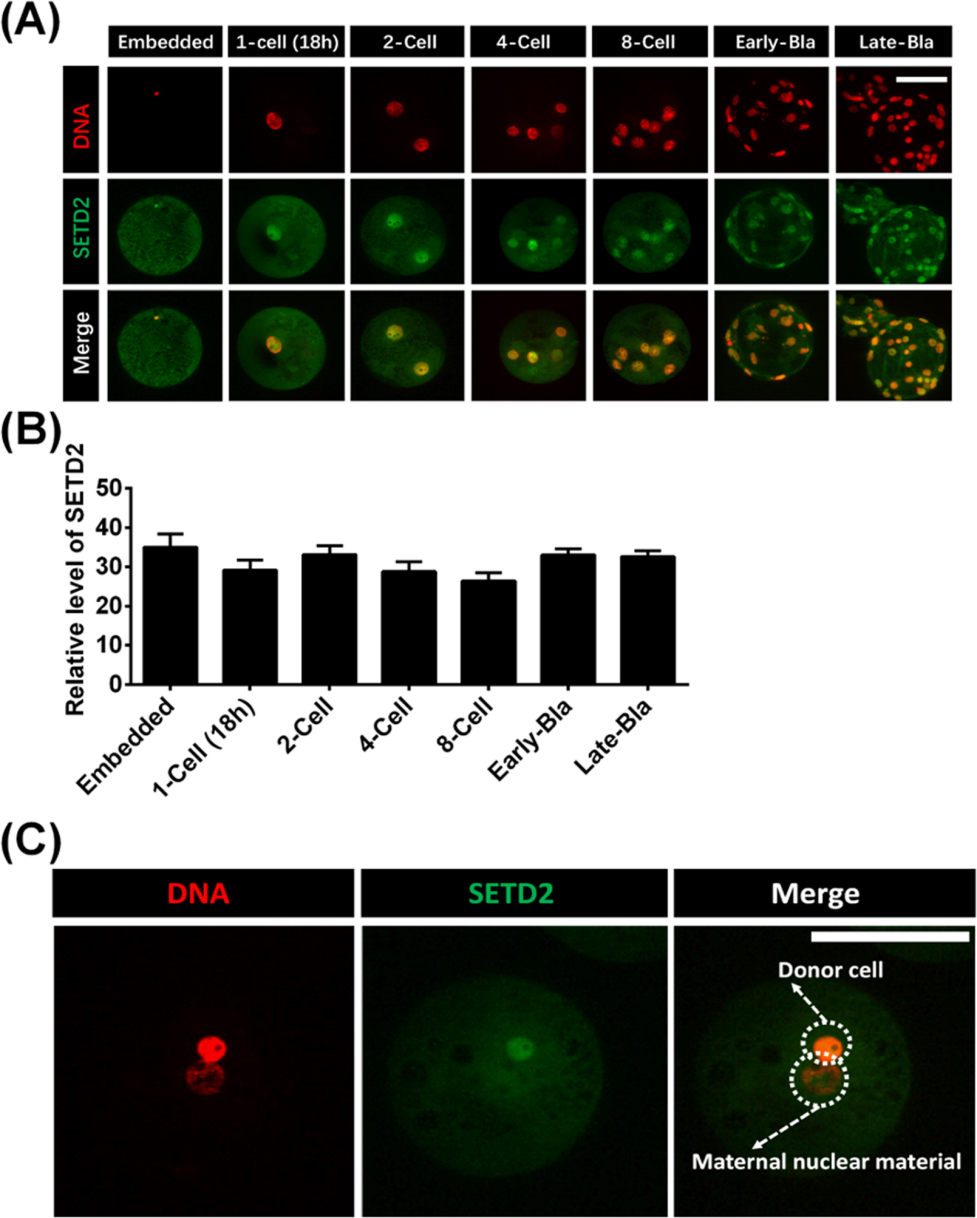
Dynamic changes of SETD2 status in porcine SCNT embryos. (A) Porcine SCNT-derived embryos were immunostained with the anti-SETD2 antibody (green), and DNA was stained with DAPI (colored red). Embedded refers to when one donor cell has been injected into the perivitelline space of an enucleated porcine MII-stage oocyte without fusion or activation. 1-Cell (1h) or (18h) indicate that reconstructed embryos were cultured for 1 or 18 h after activation. Early-Bla and Late-Bla indicate early blastocysts and late blastocysts, respectively. (B) Relative intensities of fluorescence signals from SETD2 in porcine SCNT embryos. Total of 42 SCNT embryos was analyzed in triplicate in this study. (C) The first polar body (pb1) was only removed from a MII-stage oocyte, a single donor cell was injected into the pb1-free oocyte retained MII nucleus, and electrical fusion/activation was performed. After being cultured for 18 h, the chimeric embryo was stained with the anti-SETD2 antibody. SETD2 expression was observed in the nucleus derived from the donor cell, but not in the maternal MII nucleus of the oocyte. Scale bars = 100 μm.

In contrast to our findings in IVF and PA embryos, we observed SETD2 signals throughout all stages of embryonic development in porcine SCNT embryos (Fig 4). When a single donor cell was injected into the perivitelline space of an enucleated oocyte without activation (embedded), the SETD2 signal was detectable in somatic cell (Fig 4A). After fusion, SETD2 remained at a high level comparable to that found in the embedded nucleus after culture for 18 h (1-cell stage embryo) in PZM-3 medium (Fig 4A and 4B). To clearly show that SEDT2 is not lost in restructured embryos, we injected a donor cell into a MII-stage oocyte from which the first polar body only had been removed and the MII nucleus had been retained. As shown in Fig 4C, after fusion, activation, and culture for 18 h, the nucleus derived from the donor cell was positive for the SEDT2 signal, but the maternal MII nucleus lacked this signal. These results show that SETD2 is expressed in 1-cell-stage porcine SCNT embryos, but not in comparable IVF and PA embryos.

## Discussion

SETD2 is well known as the sole H3K36 methyltransferase in yeast [22]. SETD2-dependent H3K36me3 is associated with active transcription [19], and histone H3K36 methylation is known to play important functions in transcriptional elongation, mRNA export, and suppressing cryptic transcription [23–26]. In porcine somatic cells, H3K36me3 consistently colocalizes with transcription sites that are actively synthesizing RNA (as identified by 5-fluorouridine), suggesting that histone H3K36me3 may be associated with transcription in porcine fetal fibroblasts [7]. However, no previous published study had examined the status of SETD2 in porcine somatic cells. Here, we used immunocytochemical analysis to reveal that SETD2 and H3K36me3 exhibit the same subnuclear localizations in porcine somatic cells.

SETD2, which is the mammalian ortholog of yeast SET2, has been shown to regulate H3K36me3 in human and mouse [13, 14]. Human histone H3K36 methylation is regulated by the interaction of SETD2 with hyperphosphorylated RNA polymerase II (pol II), suggesting that SETD2 may play a very important role in transcriptional regulation [8]. In the present work, SETD2 was observed in the nuclei of interphase porcine somatic cells, but not in the corresponding M-phase cells. In porcine oocytes, SETD2 signals were present in NSN-stage oocytes, but not in SN-, MI-, or MII-stage oocytes. These results suggest that the pathway through which SETD2 regulates H3K36me3 is active only in loose chromatin, where it is likely to function in transcription [27]. Given these results and information, we suggest that SETD2 may regulate H3K36me3 to play an important role in transcriptional regulation in pig.

Mammalian oocytes are arrested within ovarian follicles at the GV stage; after GVBD, the oocytes proceed to MI, extrude the first polar body, and finally arrest at MII until they are activated by fertilization or an artificial means [28]. Fully grown oocytes can resume meiosis, and the transition from the NSN to SN configuration is critical for the ability of an oocyte to acquire its full developmental capacity [29]. In the current study, we examined the localization of SETD2 during the meiotic maturation of porcine oocytes. We observed SETD2 expression in NSN-stage oocytes, but not in SN-, MI-, or MII-stage oocytes, indicating that SETD2 may be critical for the orderly progression of an oocyte through GV to MI and MII. Thus, it is suggested that the expression of SETD2 in oocytes may be regulated by a meiosis stage-dependent mechanism. It has reported that porcine SN stage oocytes are silent with transcription, while NSN stage oocytes transcribe actively [30]. Transcriptional activities shut down by means of GVBD (germinal vesicle breakdown), and the oocyte undergoes a series of chromosomal events leading to maturation [31]. In the current study, SETD2 signals did not localized in the nucleus in SN, MI, MII stages oocytes. We inferred that SETD2 may be distributed to the cytoplasm in oocytes and then redistributed to the nucleus for EGA.

Although some histone methyltransferases may methylate the same site on a histone, they tend to have slightly different functions [8]. Several other mammalian H3K36 methyltransferases have been identified in addition to SETD2 [32], including NSD1, which is a histone methyltransferase with selectivity for H3K36 and H4K20 [8, 33]. In mice, knockout of NSD1 blocked the completion of gastrulation, possibly due to cell apoptosis and mesodermal defects [33]. In humans and mice, H3K36me3 is mediated by SETD2 methyltransferase, which acts as the ortholog of yeast SET2 [13, 14, 34]. The triplicate AWS-SET-PostSET domains of SETD2 regulates the H3K36 histone methyltransferase (HMT) activity; C-terminal Set2 Rbp-1 interacting (SRI) domain mediates the interaction with RNA polymerase II (Pol II), and WW domain likely mediates protein-protein interaction [13]. Transcriptional elongation is catalyzed usually by hyperphosphorylated RNA Pol II [35]. Thus suggesting that SETD2 protein may be recruited by hyperphosphorylated RNA Pol II to the transcriptional machinery and play an important role in transcriptional regulation [8]. A previous study showed that HYPB (another name for SETD2) is required for proper patterning of histone H3K36me3 during mouse embryogenesis [13]. Embryonic gene activation (EGA) is the process by which an embryo begins to transcribe its newly formed genome. EGA is essential in order to synthesis new proteins and further cleavage to take place. EGA oocurs at different developmental stages depending on the species. In mice, EGA oocurs at 2-cell stage [36], whereas porcine embryos initiate genome transcription at the 4-cell stage [7]. In pig, H3K36me3 signals were previously observed in both PA- and IVF -derived 2-cell-stage embryos and enriched at the 4-cell stage (corresponding to the timing of EGA in pig), and the authors speculated that SETD2 or another similar methyltransferase is likely to regulate H3K36me3 modifications at the EGA stage [7]. Here, we found that, similar to the pattern of H3K36me3 previously reported in PA and IVF embryos (Figure C in S1 File), the SETD2 signal was undetectable (or very low) at the 1-cell stage, became detectable at the 2-cell stage, peaked at the 4-cell stage, and was thereafter maintained until the blastocyst stage. Based on our present results, we propose that histone H3K36me3 may be regulated by SETD2 during the preimplantation development of porcine embryos, at least prior to EGA (1- to 4-cell stages).

One important factor underlying the low developmental efficiency of somatic cell nuclear transfer (SCNT) embryos is the aberrant epigenetic reprogramming of such embryos during the early stages of development [3, 37]. Here, we found that SETD2 signals were detectable in porcine sperms. In IVF experiments, this signal was lost after fertilization (1-cell), and then was detected once more in 2-cell-stage embryos. In contrast to porcine IVF and PA embryos, however, porcine SCNT embryos exhibited SETD2 signals all the way from the 1-cell stage to the blastocyst stage. We speculate that this abnormal expression of SETD2 during porcine somatic cell reprogramming in reconstructed SCNT embryo might be one cause for the low cloning efficiency of pig.

In summary, our data indicate that SETD2 is strongly expressed during embryonic gene activation of porcine preimplantation embryos, and that it may play an important role in transcriptional regulation in embryo development. Moreover, our findings suggest that SETD2 expression in porcine SCNT embryos is abnormal as compared with that in IVF- and PA-derived embryos that could be a factor in the low efficiency of porcine cloning.

## Supporting information

S1 File (Figure A) Schematic representation of porcine oocyte maturation *in vitro*. (Figure B) Schematic representation of SCNT in current study. (Figure C) Changes in H3K36me3 in porcine IVF embryos.
(PDF)
