# Supplementary material for: Dynamic changes of SETD2, a histone H3K36 methyltransferase, in porcine oocytes, IVF and SCNT embryos
Source: PLoS One. 2018 Feb 15;13(2):e0191816. doi: 10.1371/journal.pone.0191816 (PMC5813925; doi:10.1371/journal.pone.0191816)
Supplement: S1 File — (Figure A) Schematic representation of porcine oocyte maturation in vitro. (Figure B) Schematic representation of SCNT in current study. (Figure C) Changes in H3K36me3 in porcine IVF embryos. (PDF) [file pone.0191816.s001.pdf]

Supplemental information

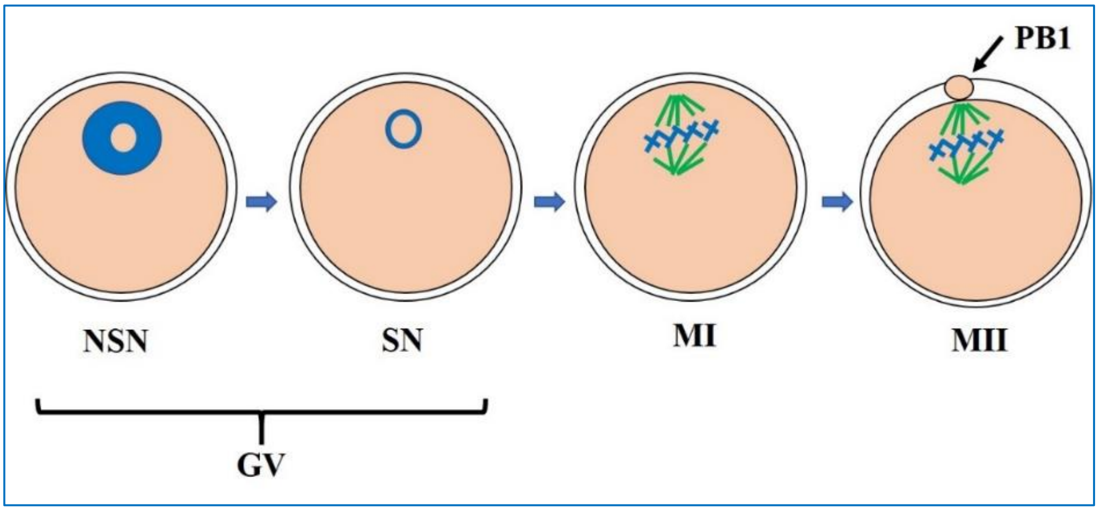

Figure A. Schematic representation of porcine oocyte maturation *in vitro*. During meiosis maturation in mammals, immature oocytes are arrested at the germinal vesicle (GV) stage. GV stage oocytes can ber matured *in vitro* in incubator. After germinal vesicle breakdown, followed by chromosome condensation (blue), spindle (green) formation (metaphase I, MI) and the first polar body (PB1) extrusion, oocytes finally arrest at the meiotic metaphase II (MII) stage until fertilization or artificial activation. However, GV stage oocytes have two kinds of chromatin configurations. Chromatin was not or less condensed around the nucleolus, termed the non-surrounded nucleolus (NSN) configuration; chromatin condensed a dense rim and around the nucleolus, termed the surrounded nucleolus (SN) configuration. The SN configuration appears after NSN with the progression of oocyte growth. In NSN stage, cells transcribe actively, and SETD2 signals concentrated on nuclei of cells. NSN-stage oocytes have more transcriptional activity and SETD2 signal than SN, MI or MII oocytes.

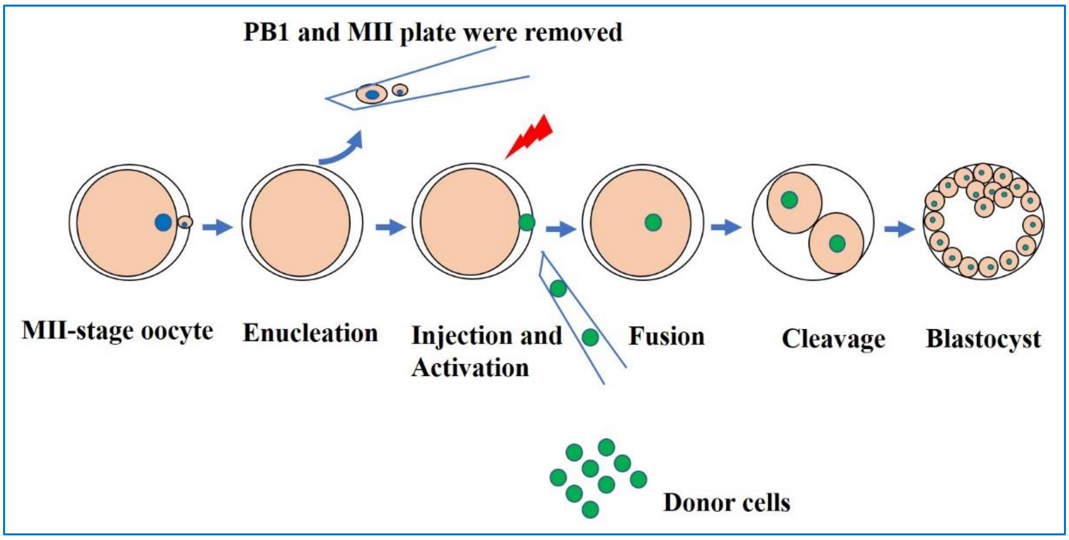

Figure B. Schematic representation of SCNT in current study. *In vitro* maturated oocytes were enucleated by aspiration of the first polar body and metaphase II plate with an enucleation needle. After enucleation, a whole donor cell was transferred into the perivitelline space of an enucleated oocyte. The reconstructed embryos were simultaneously fused and activated using BTX Electro Cell Manipulator. The reconstructed embryos were cultured in CO_2 incubator.

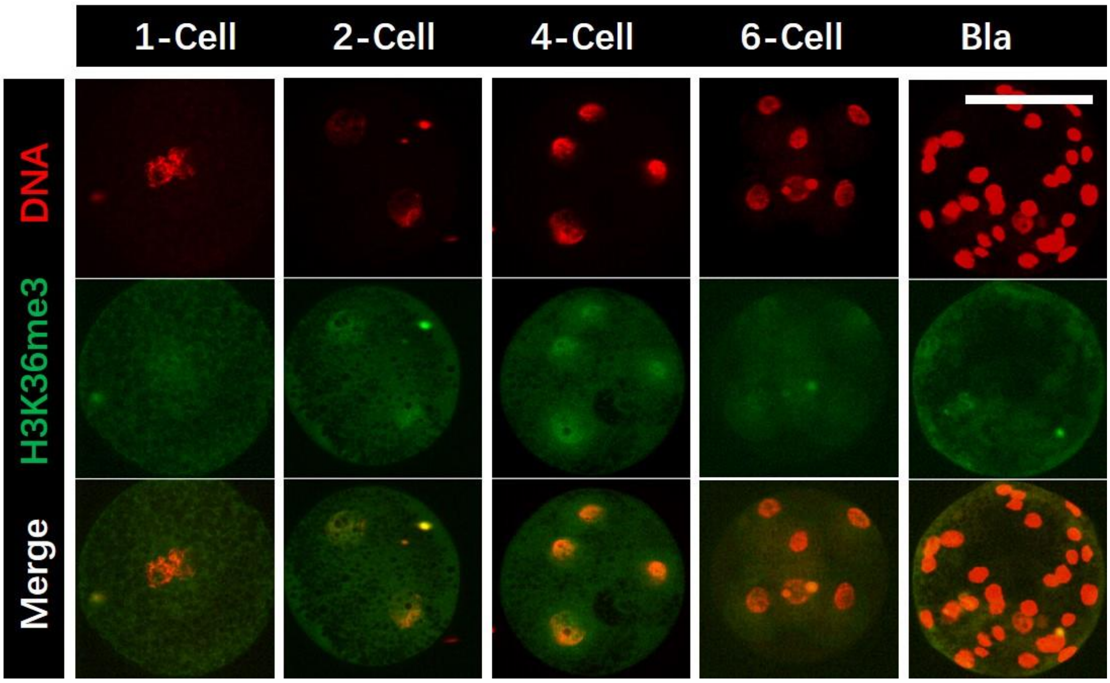

Figure C. Changes in H3K36me3 in porcine IVF embryos. Porcine IVF-derived embryos were immunostained with the anti-H3K36me3 antibody, which was visualized with an FITC-conjugated secondary antibody (green). DNA was counterstained with DAPI (colored red). Bla indicate blastocysts. Scale bars = 100 μm.
